# Untargeted metabolomic profiling reveals molecular signatures associated with type 2 diabetes in Nigerians

**DOI:** 10.1186/s13073-024-01308-5

**Published:** 2024-03-05

**Authors:** Ayo P. Doumatey, Daniel Shriner, Jie Zhou, Lin Lei, Guanjie Chen, Omolara Oluwasola-Taiwo, Susan Nkem, Adela Ogundeji, Sally N. Adebamowo, Amy R. Bentley, Mateus H. Gouveia, Karlijn A. C. Meeks, Clement A. Adebamowo, Adebowale A. Adeyemo, Charles N. Rotimi

**Affiliations:** 1 Center for Research On Genomics and Global Health, National Human Genome Research Institute, National Institutes of Health, 12 South Drive, Building 12 A, Room 1025A, Bethesda, MD 20892 USA; 2 Department of Medicine, Ring Road State Hospital, Ibadan, Nigeria; 3 Center for Bioethics & Research, Ibadan, Nigeria; 4 Department of Epidemiology and Public Health, and the Greenebaum Comprehensive Cancer Center, University of Maryland School of Medicine, Baltimore, MD USA

**Keywords:** Metabolomics, Type 2 diabetes, Africans, Biomarkers

## Abstract

**Background:**

Type 2 diabetes (T2D) has reached epidemic proportions globally, including in Africa. However, molecular studies to understand the pathophysiology of T2D remain scarce outside Europe and North America. The aims of this study are to use an untargeted metabolomics approach to identify: (a) metabolites that are differentially expressed between individuals with and without T2D and (b) a metabolic signature associated with T2D in a population of Sub-Saharan Africa (SSA).

**Methods:**

A total of 580 adult Nigerians from the Africa America Diabetes Mellitus (AADM) study were studied. The discovery study included 310 individuals (210 without T2D, 100 with T2D). Metabolites in plasma were assessed by reverse phase, ultra-performance liquid chromatography and mass spectrometry (RP)/UPLC-MS/MS methods on the Metabolon Platform. Welch’s two-sample t-test was used to identify differentially expressed metabolites (DEMs), followed by the construction of a biomarker panel using a random forest (RF) algorithm. The biomarker panel was evaluated in a replication sample of 270 individuals (110 without T2D and 160 with T2D) from the same study.

**Results:**

Untargeted metabolomic analyses revealed 280 DEMs between individuals with and without T2D. The DEMs predominantly belonged to the lipid (51%, 142/280), amino acid (21%, 59/280), xenobiotics (13%, 35/280), carbohydrate (4%, 10/280) and nucleotide (4%, 10/280) super pathways. At the sub-pathway level, glycolysis, free fatty acid, bile metabolism, and branched chain amino acid catabolism were altered in T2D individuals. A 10-metabolite biomarker panel including glucose, gluconate, mannose, mannonate, 1,5-anhydroglucitol, fructose, fructosyl-lysine, 1-carboxylethylleucine, metformin, and methyl-glucopyranoside predicted T2D with an area under the curve (AUC) of 0.924 (95% CI: 0.845–0.966) and a predicted accuracy of 89.3%. The panel was validated with a similar AUC (0.935, 95% CI 0.906–0.958) in the replication cohort. The 10 metabolites in the biomarker panel correlated significantly with several T2D-related glycemic indices, including Hba1C, insulin resistance (HOMA-IR), and diabetes duration.

**Conclusions:**

We demonstrate that metabolomic dysregulation associated with T2D in Nigerians affects multiple processes, including glycolysis, free fatty acid and bile metabolism, and branched chain amino acid catabolism. Our study replicated previous findings in other populations and identified a metabolic signature that could be used as a biomarker panel of T2D risk and glycemic control thus enhancing our knowledge of molecular pathophysiologic changes in T2D. The metabolomics dataset generated in this study represents an invaluable addition to publicly available multi-omics data on understudied African ancestry populations.

**Supplementary Information:**

The online version contains supplementary material available at 10.1186/s13073-024-01308-5.

## Background

Type 2 diabetes (T2D) is a public health threat, affecting 463 million people worldwide in 2019 and is projected to affect 700 million by 2045 [1]. Low- and middle-income countries are expected to see the largest increase in T2D incidence in the coming years [1, 2]. For example, Sub-Saharan Africa (SSA) is predicted to have the highest increase of any geographic region at 129%, reaching 55 million by 2045 [3]. The increase appears to be driven by the sustained increase in obesity prevalence [4]. The twin epidemiology of T2D and obesity termed “diabesity” has been associated with sedentary lifestyles, calorie-dense diets, and environmental factors in high-income countries [5–7]. Epidemiology studies in SSA have linked the increase in T2D with the growing adoption of a westernized lifestyle [8–10]. However, studies to understand the cellular and molecular basis of T2D in SSA are scarce. Molecular mechanisms such as oxidative stress, inflammation, or shortening of telomeres have been associated with the pathophysiology of T2D, either contributing to or co-occurring with impairment in glucose metabolism pathways [11–17]. These findings emerged from studies that used a variety of omics technologies including genomics, transcriptomics, proteomics, epigenomics, and most recently metabolomics [18–22].

Metabolomics is the study of the metabolism and metabolites in an organism. It includes the detection of thousands of small endogenous and exogenous molecules (< 1000 Da) in biofluids and other biospecimens [23]. Metabolomics can connect genes and environmental factors by capturing the output of the genome but also the input from the environment including drugs and food [24]. The ability of metabolomics to systematically capture endogenous and exogenous metabolites makes it an attractive investigative tool to help understand the relative roles of multiple factors in disease states. As such, the metabolome is considered a better reflection of a given phenotype than data from other omics approaches [24, 25]. Additionally, it has been proposed that metabolomics can capture gene-environment interactions, a component of the missing heritability observed in genomic studies [26]. Against this background, metabolomic studies have been conducted to better understand the pathophysiology of various disorders including cancer, infectious diseases, and cardiometabolic diseases [27–33]. These studies have been predominantly conducted in model organisms (primarily murine models) or in human populations from Europe, North America, and Southeast Asia [34–39]. Studies of understudied populations (including populations from Africa) have the potential to provide insights into metabolic pathways that may be differentially involved in molecular mechanisms of various diseases, including T2D.

In African populations, metabolomic studies have been overwhelmingly used in infectious diseases such as tuberculosis for novel biomarker discovery, disease characterization or to understand mechanistic processes involved in disease development and progression [40–44]. Outside of infectious diseases, metabolomic studies in Africa have been performed in the context of pediatric malnutrition and newborn screening [45, 46]. Few studies in SSA have attempted to investigate metabolic signatures associated with metabolic diseases such as obesity and T2D [47–49]. For example, Dugas and co-authors compared serum metabolic profiles of 69 African American women with 97 South African and 82 Ghanaian women, and found a shared obesity-associated amino acid metabolite profile between African Americans and South Africans as well as site-specific obesity-associated metabolites, suggesting the effect of the local environment on the phenotype [48]. A metabolomic study of glucose tolerance and T2D in a prospective cohort of 75 Black South African women showed that certain metabolite patterns in lysophospholipid metabolism, bile acid pool, and amino acid catabolism can be useful to identify and monitor T2D risk prior to disease onset [49]. These studies were limited by two main factors: small sample size and small metabolite panels.

To our knowledge, no metabolomics study has been conducted in Nigeria despite the high burden of prediabetes and diabetes in the last decade [50, 51]. Additionally, one of our previous studies in Nigerians has reported that patients with T2D have an atypical metabolic presentation characterized by both insulin resistance and reduced insulin secretion [52], but the molecular characteristics that may be involved in these changes are unknown. Thus, the implementation of metabolomics study in this population could help understand observed metabolic features. Also, studying Nigerians, a population in nutritional transition like populations in many other low-to-moderate income countries with similar environmental factors, will give us not only a comprehensive snapshot of the metabolic changes associated with T2D but will also provide data for comparison with similar populations in SSA.

In the present study, we conducted an untargeted metabolomic study in a cohort of well-phenotyped adult Nigerians from the long-running Africa America Diabetes Mellitus (AADM) Study. Using data obtained on over 1000 plasma metabolites profiled on the Metabolon platform, we compared the metabolomic profiles in individuals with and without T2D. Our goals included the identification of key metabolites and metabolic pathways associated with T2D. Further, we searched for metabolic signature associated with T2D in independent discovery and replication samples. Findings from this largest metabolomic study in Africa hold the potential to providing insights into the metabolic dysregulation associated with T2D.

## Methods

### Study participants

The parent study, the Africa America Diabetes Mellitus study (AADM), is a long-standing genetic epidemiology study of T2D and other cardiometabolic traits, enrolled participants from multiple medical centers in Nigeria, Ghana, and Kenya [53]. Participants in this metabolomics study were selected from the AADM longitudinal sub-study of 650 participants enrolled from a single study site in Ibadan, Nigeria, for deep phenotyping in order to better characterize multiple cardiometabolic traits in an urban setting [54]. A sample of 310 participants was randomly selected for the discovery sample without conditioning on any specific phenotype. The remaining 270 participants who had plasma samples that met the requirements of the metabolomics workflow were studied as the replication study. Most of the participants (96.5%) included in the present were members of the Yoruba ethnic group. Demographic information was collected using standardized questionnaires. Anthropometric measurements, medical history and clinical biomarkers were obtained by trained study staff during a clinic visit. Weight was measured in light clothes on an electronic scale to the nearest 0.1 kg and height was measured with a stadiometer to the nearest 0.1 cm. Body mass index (BMI) was computed as weight (kg) divided by the square of height in meters (m^2^). T2D status was determined using the American Diabetes Association (ADA) criteria of fasting plasma glucose cut-off of ≥ 7.0 mmol/L (126 mg/dL), combined with either a 2-h post load value of ≥ 11.1 mmol/L (200 mg/dL) on an oral glucose tolerance test (OGTT) or with taking glucose-lowering medication as prescribed by a physician. Blood samples were drawn from each participant after at least an 8-h overnight fast. Clinical chemistry (including glucose, insulin, and lipids) was assayed on fasting samples using COBAS® autoanalyzer systems (Roche Diagnostics, Indianapolis, Indiana) following the manufacturer’s instructions. Homeostatic model assessment for insulin resistance (HOMA—IR) was calculated using the following formula: fasting glucose (mmol/L) X fasting insulin (µU/L) / 22.5).

### Untargeted plasma metabolomics

#### Sample preparation and Ultrahigh Performance Liquid Chromatography-Tandem Mass Spectroscopy (UPLC-MS/MS)

Untargeted metabolomic data were obtained using well established protocols at Metabolon Inc. (Metabolon, Inc., Morrisville, NC, USA) as previously described [55, 56]. Prior to sample extraction, several recovery standards were added to samples for quality control (QC) purposes. All plasma samples (both the discovery and replication samples) were treated with aqueous methanol to remove proteins; resulting extracts were divided into 5 fractions: two for analysis by two separate reverse phase (RP), Ultra Performance, Liquid Chromatography (UPLC), Mass Spectrometry (MS), (RP)/UPLC-MS/MS methods with positive ion mode electrospray ionization (ESI), one for analysis by RP/UPLC-MS/MS with negative ion mode ESI, one for analysis by hydrophilic interaction liquid chromatography (HILIC), HILIC/UPLC-MS/MS with negative ion mode ESI, and one fraction reserved for backup. All methods used a Waters ACQUITY ultra-performance liquid chromatography (UPLC) and a Thermo Scientific Q-Exactive high resolution/accurate mass spectrometer interfaced with a heated electrospray ionization (HESI-II) source and Orbitrap mass analyzer operated at 35,000 mass resolution. The detailed description of the liquid chromatography-gas chromatography (LC-GC) was previously published [55–57].

#### Data extraction, compound identification and curation

Raw data were extracted, peak-identified and QC processed using Metabolon’s hardware and software. Compounds were identified by comparison to library entries of purified standards or recurrent unknown entities. Metabolon maintains a library based on authenticated standards that contains the retention time/index (RI), mass to charge ratio (m/z), and chromatographic data (including MS/MS spectral data) on all molecules present in the library. Furthermore, biochemical identifications are based on three criteria: retention index within a narrow RI window of the proposed identification, accurate mass match to the library ± 10 ppm, and the MS/MS forward and reverse scores between the experimental data and authentic standards. The MS/MS scores are based on a comparison of the ions present in the experimental spectrum to the ions present in the library spectrum. While there may be similarities between these molecules based on one of these factors, the use of all three data points can be utilized to distinguish and differentiate biochemicals. More than 3300 commercially available purified standard compounds have been acquired and registered for analysis for determination of their analytical characteristics. Additional mass spectral entries have been created for structurally unnamed biochemicals, which have been identified by virtue of their recurrent nature (both chromatographic and mass spectral).

A variety of curation procedures were carried out to ensure that a high-quality dataset was available for statistical analysis and data interpretation. The QC and curation processes were designed to ensure accurate and consistent identification of true chemical entities, and to remove those representing system artifacts, mis-assignments, and background noise. Metabolon data analysts use proprietary visualization and interpretation software to confirm the consistency of peak identification among the various samples. Library matches for each compound were checked for each sample and corrected if necessary.

Peaks were quantified using the area under the receiver operating characteristic (ROC) curve (AUC). For studies spanning multiple days, a data normalization step was performed to correct for variation resulting from instrument inter-day tuning differences. Essentially, each compound was corrected in run-day blocks by registering the medians to equal one (1.00) and normalizing each data point proportionately. After batch-normalization of the data, missing values were imputed using the minimum observed method i.e., for each metabolite, the missing values were replaced with its observed minimum. This imputation method was chosen based on simulation studies comparing it to other methods based on type I error and power for the two-sample t-test. The batch-normalized imputed data was then transformed using the natural log and used for downstream analyses [58]

### Statistical analysis

Anthropometric and clinical variables were analyzed using *SAS/STAT* software (version 9.4). Most anthropometric and clinical variables in this study are not normally distributed and are therefore summarized by medians and interquartile ranges (IQR). To compare medians between individuals with T2D and those without T2D, we performed a non-parametric test (the two-sample median test) using the NPAR1WAY procedure in *SAS*.

To identify differentially expressed metabolites (DEMs) between individuals with T2D and those without T2D, we conducted Welch’s two-sample t-test with nominal significance defined as *p* < 0.05 and adjusted significance for multiple comparisons as a false discovery rate (FDR) *q* < 0.10. We also conducted a classification test using a random forest (RF) algorithm to identify a set of metabolites/biomarkers that can accurately classify individuals with and without T2D. RF is an unbiased and supervised machine learning method based on decision trees [59]. The multivariable biomarker discovery analysis was performed in MetaboAnalyst 5.0 [60]. All other statistical analyses and data visualizations were performed in ArrayStudio, JMP or the R statistical environment, R package (version 4.0.5) (http://cran.r-project.org/) [61]

For the multivariable biomarker discovery analysis, the filtered, batch-normalized, imputed and log transformed peak intensity data table was uploaded into MetaboAnalyst 5.0. [58]. T2D status (Yes/No) was used as the binomial outcome and individuals without T2D as the reference category. Receiver operating characteristics (ROC) curves were generated by Monte Carlo cross-validation using balanced subsampling. In each iteration, 2/3 of the samples were used to evaluate feature importance and the remaining 1/3 were used to validate the models generated. The top-ranking features based on importance were used to construct the classification models. The process is repeated several times to calculate the performance and confidence intervals of each model. Using the predictive accuracy of the biomarker models generated, we retained the biomarker model with the highest predictive accuracy for downstream analyses. For the evaluation of the biomarker model retained in the discovery phase, we used the ROC curve-based model creation and evaluation option of MetaboAnalyst 5.0 which permits the manual selection of any combination of features to create a biomarker model. We manually selected the metabolites included in the biomarker model retained in the discovery phase and similarly used the RF algorithm to evaluate the ability of these biomarkers to predict T2D cases and controls among the 270 samples of the replication cohort. To assess the relationships between metabolites in the identified biomarker panel and key clinical indexes of T2D, we conducted a correlation analysis (Spearman correlation) using *SAS/STAT* (version 9.4).

## Results

### Characteristics of the participants in the discovery study

Individuals with T2D were significantly older and had a larger waist circumference than those without T2D (Table 1). Markers of glycemic status, including plasma glucose, insulin, HOMA-IR, and HbA1c, were significantly higher in individuals with T2D compared to those without T2D, despite 97% of individuals with T2D being on treatment with oral hypoglycemic agents. This finding indicates poor glycemic control in these individuals (Table 1, Additional file 1 (Table S1A)). Metformin (Met) and sulfonylureas (SU) were the commonly used treatments either as monotherapy (Met only or SU only) or bitherapy (Met + SU) (Additional file 1 (Table S1A)). Of the lipids examined, triglycerides levels were significantly higher in T2D cases than controls (Table 1).

**Table 1:** Anthropometric and clinical characteristics of the discovery cohort

Variable	Overall (*n* = 310)	T2D (*n* = 100)	Non-T2D (*n* = 210)	*P*-value^*^
Age (Years)	56.2 (17.7)	60.2 (11.9)	54.4(18.6)	0.0007^¥^
% Male	22.6	18	24.8	0.18
BMI (kg/m^2^)	30.5 (8.77)	32.6 (9.02)	30.2 (8.8)	0.15
PFM	38.9 (12.9)	40.5 (12.9)	38.1 (13.0)	0.1
Waist circumference (cm)	99.0 (16.0)	101.5 (16.00)	98.0 (16.50)	0.02^¥^
WHR	0.96 (0.07)	0.96 (0.10)	0.95 (0.08)	0.23
Glucose (mg/dl)	87.0 (21.0)	118.5 (62.00)	82.5 (13.00)	< 0.0001^¥^
Insulin (μU/mL)	7.8 (6.8)	9.2 (7.5)	7.2 (5.90)	0.008^¥^
HOMA-IR	1. 7 (1.9)	2.7 (3.00)	1.5 (1.3)	< 0.0001^¥^
Hba1c (%)	5.6 (1.1)	7.4 (2.6)	5.4 (0.50)	< 0.0001^¥^
Total cholesterol (mg/dl)	193.0 (73)	200.0 (75.5)	191.5 (71.5)	0.17
HDL-Cholesterol (mg/dl)	49.8 (20.4)	50.6 (23.9)	48.1 (19.8)	0.22
LDL-Cholesterol (mg/dl)	120.0 (64.0)	119.0 (60.5)	120.5 (66.0)	0.87
Triglycerides (mg/dl)	91.0 (47.0)	105.5 (65.5)	86.0 (45.5)	< 0.0001^¥^

Data is displayed as median (interquartile range)

Ratio compared by chi-square test

*BMI* Body mass index, *PFM* Percentage fat mass, *WHR* Waist-to-hip ratio, *HbA1c* Hemoglobin A1c, *TG* Triglycerides, *HDL* High density lipoproteins, *LDL* Low density lipoproteins, *T2D* Individuals with T2D, *Non-T2D* Individuals without T2D

^*^Medians were compared using the two-sample median test

^¥^denotes statistically different medians

### Overall profiling of differently expressed metabolites (DEM) in individuals with T2D

**Fig. 1:**
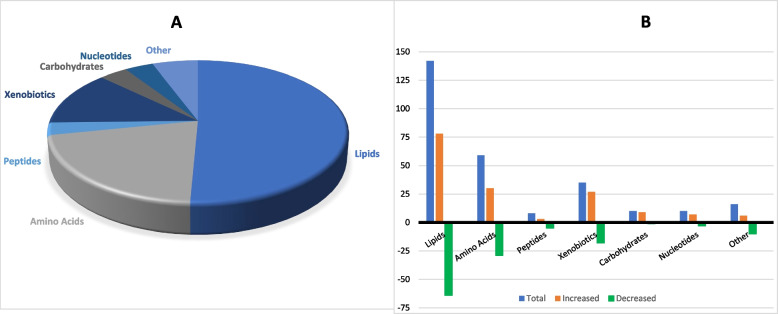
Classification of differentially expressed metabolites in T2D by super pathways. **A** Pie chart of super pathways associated with differentially expressed metabolites. **B** Number of differentially expressed metabolites in T2D by super pathways. Y-axis represents the number of metabolites

A total of 1116 metabolites or compounds of known identity were identified in the 310 plasma samples of the discovery phase samples (Additional file 2 (Table S2A)). At a nominal point-wise significance level of 0.05, 301 metabolites were significantly different between individuals with and without T2D (Additional file 2 (Table S2B)). After adjusting for multiple testing (FDR < 0.1), 280 out of the 301 metabolites remained differentially expressed in T2D individuals compared with those without T2D, including 156 metabolites that were increased and 124 that were decreased in T2D (Fig. 1A, Additional file 2 (Table S2C)). Overall, these metabolites predominantly belong to the super pathway lipids (51%), amino acids (21%), xenobiotics (13%), carbohydrates (4%) and nucleotides (4%) (Fig. 1B).

**Fig. 2:**
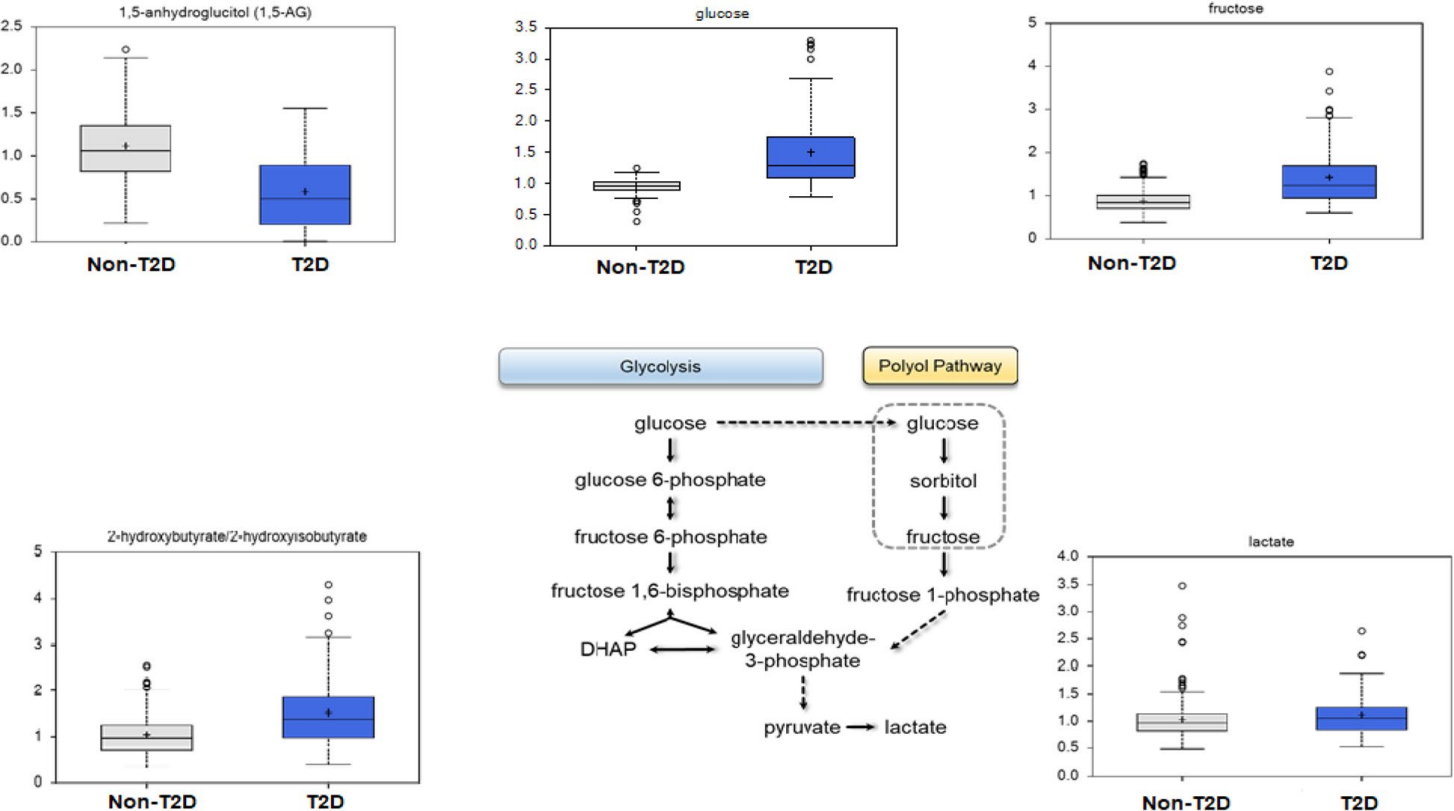
Box Plots of differentially expressed metabolites in the carbohydrate super pathway (glucose utilization) and associated metabolism pathways

The top metabolites differentially expressed in individuals with T2D sorted based on fold change (FC) and FDR < 0.10 are shown in Table 2 (DEMs upregulated with respect to T2D) and Table 3 (DEMs downregulated with respect to T2D). Glucose was increased (FC = 1.56) while key components of glucose utilization, especially glycolysis, gluconeogenesis, and pyruvate metabolism including 1,5-anhydroglucitol (1,5 AG), were decreased (FC = 0.52) in individuals with T2D (Table 3, Fig. 2). As expected, anti-diabetic drugs (classified as xenobiotics) used by most treated participants with T2D (metformin, FC = 20.27; pioglitazone, FC = 6.12; gliclazide, FC = 2.58) (Table 2, Additional file 1 (Table S1B)) were among the DEMs. There was a marginally higher lactate level (a marker of glucose utilization) in individuals with T2D (Fig. 2). Additionally, mannose (FC = 1.98) and fructose (FC = 1.62) were both increased in individuals with T2D. Fructose can be derived from the diet or be produced in vivo from glucose through the polyol pathway (Fig. 2). 2-hydroxybutyrate, a known insulin resistance marker, was also significantly higher in individuals with T2D compared with those without T2D (Fig. 2). Several of the top DEMs were associated with different lipids sub-pathways including fatty acid metabolism (medium chain fatty acid [5-dodecenoate] and long chain monounsaturated fatty acid [myristoleate, palmitoleate]), as well as progestin and pregnenolone steroids, which were all decreased in T2D (Tables 2 and 3).

**Table 2:** The most significantly upregulated metabolites in T2D based on fold change

Super Pathway	Sub Pathway	Biochemical Name	KEGG	HMDB	FC (T2D vs non-T2D)	*p*-value	q-value
Xenobiotics	Drug—Metabolic	glyburide	C07022	HMDB0015151	**1.55**	0.0127	0.0482
Carbohydrate	Glycolysis, Gluconeogenesis, and Pyruvate Metabolism	glucose	C00031	HMDB0000122	**1.56**	0.0000	0.0000
Lipid	Fatty Acid Metabolism (Acyl Carnitine, Hydroxy)	(R)-3-hydroxybutyrylcarnitine		HMDB0013127	**1.56**	0.0005	0.0057
Amino Acid	Lysine Metabolism	Fructosyl-lysine		HMDB0034879	**1.59**	0.0000	0.0000
Xenobiotics	Food Component/Plant	gluconate	C00257	HMDB0000625	**1.60**	0.0000	0.0000
Xenobiotics	Food Component/Plant	ferulic acid 4-sulfate		HMDB0029200	**1.60**	0.0107	0.0446
Lipid	Fatty Acid, Monohydroxy	5-hydroxyhexanoate		HMDB0000525	**1.61**	0.0002	0.0034
Carbohydrate	Fructose, Mannose and Galactose Metabolism	fructose	C00095	HMDB0000660	**1.62**	0.0000	0.0000
Amino Acid	Leucine, Isoleucine and Valine Metabolism	1-carboxyethylisoleucine			**1.64**	0.0000	0.0000
Amino Acid	Tyrosine Metabolism	m-tyramine sulfate			**1.66**	0.0286	0.0777
Cofactors and Vitamins	Hemoglobin and Porphyrin Metabolism	L-urobilin	C05793	HMDB0004159	**1.81**	0.0011	0.0093
Xenobiotics	Food Component/Plant	mannonate			**1.88**	0.0000	0.0000
Lipid	Secondary Bile Acid Metabolism	glycodeoxycholate	C05464	HMDB00631	**1.90**	0.0112	0.0453
Carbohydrate	Fructose, Mannose and Galactose Metabolism	mannose	C00159	HMDB0000169	**1.98**	0.0000	0.0000
Xenobiotics	Xanthine Metabolism	1,7-dimethylurate	C16356	HMDB0011103	**2.00**	0.0081	0.0379
Lipid	Primary Bile Acid Metabolism	glycocholate	C01921	HMDB0000138	**2.01**	0.0168	0.0563
Xenobiotics	Xanthine Metabolism	1,3,7-trimethylurate	C16361	HMDB0002123	**2.02**	0.0246	0.0725
Lipid	Secondary Bile Acid Metabolism	deoxycholic acid 12-sulfate			**2.05**	0.0003	0.0039
Lipid	Secondary Bile Acid Metabolism	taurodeoxycholate	C05463	HMDB0000896	**2.54**	0.0027	0.0180
Xenobiotics	Drug—Metabolic	Gliclazide			**2.58**	0.0026	0.0173
Xenobiotics	Chemical	1,2,3-benzenetriol sulfate (2)		HMDB0060018	**2.66**	0.0318	0.0817
Xenobiotics	Food Component/Plant	saccharin	C12283	HMDB0029723	**3.34**	0.0350	0.0890
Lipid	Primary Bile Acid Metabolism	taurocholate	C05122	HMDB0000036	**4.51**	0.0172	0.0571
Xenobiotics	Drug—Metabolic	pioglitazone	C07675	HMDB0015264	**6.12**	0.0049	0.0270
Xenobiotics	Drug—Metabolic	metformin	C07151	HMDB0001921	**20.27**	0.0000	0.0000

**Table 3:** The most significantly downregulated metabolites in T2D based on fold change

Super Pathway	Sub Pathway	Biochemical Name	KEGG	HMDB	FC (T2D vs non-T2D)	*p*-value	q-value
Xenobiotics	Food Component/Plant	ethyl alpha-glucopyranoside		HMDB0029968	**0.30**	0.0008	0.0070
Xenobiotics	Food Component/Plant	(2,4 or 2,5)-dimethylphenol sulfate			**0.39**	0.0338	0.0862
Partially Characterized Molecules	Partially Characterized Molecules	branched-chain, straight-chain, or cyclopropyl 10:1 fatty acid (1)			**0.51**	0.0000	0.0000
Carbohydrate	Glycolysis, Gluconeogenesis, and Pyruvate Metabolism	1,5-anhydroglucitol (1,5-AG)	C07326	HMDB0002712	**0.52**	0.0000	0.0000
Lipid	Progestin Steroids	5alpha-pregnan-3beta,20alpha-diol monosulfate (2)			**0.55**	0.0085	0.0389
Lipid	Progestin Steroids	5alpha-pregnan-3beta-ol,20-one sulfate			**0.58**	0.0255	0.0743
Lipid	Progestin Steroids	5alpha-pregnan-3beta,20beta-diol monosulfate (1)		HMDB0240580	**0.60**	0.0159	0.0548
Amino Acid	Urea cycle; Arginine and Proline Metabolism	N-methylproline		HMDB0094696	**0.61**	0.0170	0.0568
Lipid	Fatty Acid Metabolism (Acyl Carnitine, Monounsaturated)	5-dodecenoylcarnitine (C12:1)		HMDB13326	**0.62**	0.0000	0.0005
Lipid	Progestin Steroids	pregnanediol-3-glucuronide		HMDB0010318	**0.62**	0.0066	0.0332
Lipid	Progestin Steroids	pregnanolone/allopregnanolone sulfate	C05480	HMDB0240591	**0.62**	0.0209	0.0665
Lipid	Pregnenolone Steroids	17alpha-hydroxypregnanolone glucuronide			**0.69**	0.0024	0.0170
Nucleotide	Pyrimidine Metabolism, Orotate containing	dihydroorotate	C00337	HMDB03349	**0.69**	0.0035	0.0220
Lipid	Androgenic Steroids	5alpha-androstan-3alpha,17alpha-diol monosulfate		HMDB0000412	**0.69**	0.0090	0.0399
Partially Characterized Molecules	Partially Characterized Molecules	glutamine_degradant			**0.70**	0.0000	0.0008
Lipid	Medium Chain Fatty Acid	5-dodecenoate (12:1n7)		HMDB0000529	**0.71**	0.0003	0.0039
Lipid	Fatty Acid, Dicarboxylate	decadienedioic acid (C10:2-DC)			**0.71**	0.0399	0.0971
Lipid	Long Chain Monounsaturated Fatty Acid	myristoleate (14:1n5)	C08322	HMDB0002000	**0.72**	0.0004	0.0047
Amino Acid	Alanine and Aspartate Metabolism	N, N-dimethylalanine			**0.73**	0.0000	0.0007
Peptide	Gamma-glutamyl Amino Acid	gamma-glutamylglutamine	C05283	HMDB0011738	**0.73**	0.0143	0.0510
Lipid	Long Chain Monounsaturated Fatty Acid	palmitoleate (16:1n7)	C08362	HMDB0003229	**0.73**	0.0218	0.0680
Partially Characterized Molecules	Partially Characterized Molecules	bilirubin degradation product, C17H20N2O5 (2)			**0.74**	0.0129	0.0485
Lipid	Fatty Acid Metabolism (Acyl Carnitine, Dicarboxylate)	pimeloylcarnitine/3-methyladipoylcarnitine (C7-DC)			**0.74**	0.0178	0.0582
Nucleotide	Purine Metabolism, Adenine containing	adenosine 5'-diphosphate (ADP)	C00008	HMDB0001341	**0.74**	0.0222	0.0688
Amino Acid	Glutamate Metabolism	glutamine	C00064	HMDB0000641	**0.75**	0.0047	0.0270

In the replication study, we evaluated DEMs in T2D in an additional 270 participants from the AADM study. Like in the discovery cohort, the participants with T2D in the replication cohort were older and had significantly higher glucose, HOMA-IR, HbA1c, and insulin than those without T2D (Additional file 1 (Table S3)). The total number of metabolites identified in the replication cohort was slightly lower compared to the total number of metabolites identified in the discovery phase cohort (1071 vs. 1116 metabolites) while DEMs is higher (343 vs. 280) (Additional file 3 (Table S4A/B)). The majority of DEMs belong to the super pathways of lipids (51%), amino acids (20%), xenobiotics (11.1%) and carbohydrates (4.6%). The super pathways represented by the DEMs were similar in both discovery and replication cohorts (Additional file 3 (Table S4C), Additional file 4 (Fig S1)). One hundred-forty-one (141) of the 280 DEMs identified in the discovery cohort were also DEMs in the replication cohort (Additional file 3 (Table S4D)).

### Fatty acid and bile acid metabolisms are among altered pathways in T2D

**Fig. 3:**
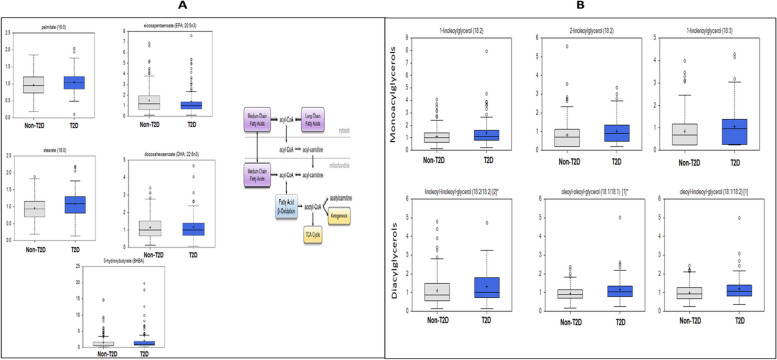
Examples of differentially expressed lipids in T2D and associated metabolism pathways. **A**. DEMs in fatty acid metabolism pathways (free fatty acids: from upper left to lower left, palmitate, eicosapentaenoate (EPA;20:5n3), stearate, docohexaenoate (DHA;22:6n3), 3-hydroxybutyrate (BHBA); far right: fatty acid metabolism implicating FFA differentially expressed in this study. **B**. Examples of differentially expressed monoacylglycerols and diacylglycerols (products of lipolysis) in T2D. Monoacylglycerols: Left to right, 1-linoleoylglycerol (18:2); 2-linoleoylglycerol (18:2); 1-linoleoyglycerol (18:3). Diacylglycerols: Left to right, linoleoyl- linoleoyl-glycerol (18:2/18:2); oleoyl- oleoyl-glycerol (18:1/18:1); oleoyl-linoleoyl-glycerol (18:1/18:2)

Overall, metabolites in the lipids super pathway were among the most statistically significant DEMs between individuals with T2D and those without T2D. These metabolites include plasma free fatty acids (FFA) such as stearate (FC = 1.13), margarate (FC = 1.20), adrenate (FC = 1.22), and palmitate (nominally higher in T2D, FC = 1.05, *p* = 0.05) that were higher in individuals with T2D compared with those without T2D (Fig. 3A, Additional file 2 (Table S2D)). Additionally, both diacylglycerols and monoacylglycerols, downstream products of triglyceride degradation, were significantly higher in individuals with T2D (Fig. 3B, Additional file 2 (Table S2D)). To further investigate the source of the high levels of FFA, we analyzed by-products of fatty acid oxidation, especially carnitine derivatives that have been reported to be high in T2D cases in other populations. We found no statistical differences in short-chain acyl carnitines between the two groups in this study (Additional file 4 (Fig S2)). Additionally, monounsaturated and polyunsaturated acyl carnitines were generally lower in individuals with T2D compared to those without T2D (5-dodecenoylcarnitine, FC = 0.62; arachidonoylcarnitine, FC = 0.77) (Table 2, Additional file 2 (Table S2D)). Interestingly, ω-oxidation, an alternative to β-oxidation, appeared to be increased. In fact, ω-oxidation end products such as 3-hydroxyadipate (FC = 1.36) and 3-hydroxydodecanedioate (FC = 1.29) were higher in T2D cases compared to controls (Additional file 2 (Table S2D)). The largely diet-derived eicosapentaenoate (EPA) and docosahexaenoate (DHA) were not significantly higher in T2D individuals (Fig. 3A).

**Fig. 4:**
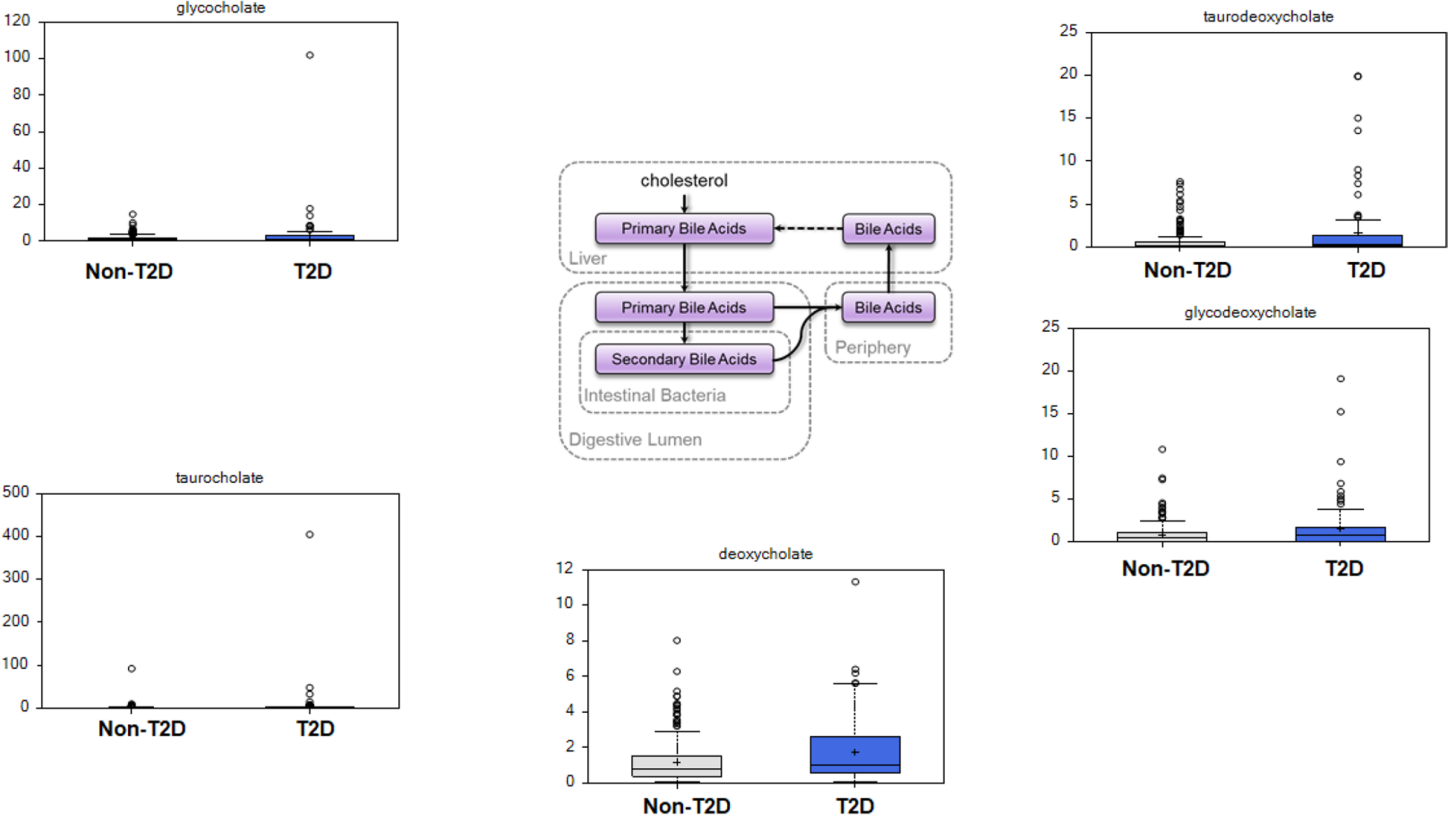
Box plots of examples of differentially expressed metabolites in the primary and secondary bile acid synthesis metabolisms. Left panel: primary bile acids: glycocholate and taurocholate are increased in individuals with T2D compared to those without T2D. Middle panel: top diagram represents the primary and secondary bile acid synthesis pathway in the liver and the digestive lumen; the bottom represents the box plot of deoxycholate concentrations in individuals with T2D and without T2D. Right panel: Secondary bile acids, taurodeoxycholate and glycodeoxycholate are increased in individuals with T2D compared to those without T2D

Bile acids, also members of the lipid super pathway and known for their associations with insulin resistance and the development of T2D, were significantly increased in individuals with T2D compared to those without T2D. These bile acids include the primary bile acids glycocholate and taurocholate as well as the secondary bile acids deoxycholate, glycodeoxycholate, and taurodeoxycholate (Fig. 4).

### Branched chain amino acids (BCAA) are significantly increased in T2D

**Fig. 5:**
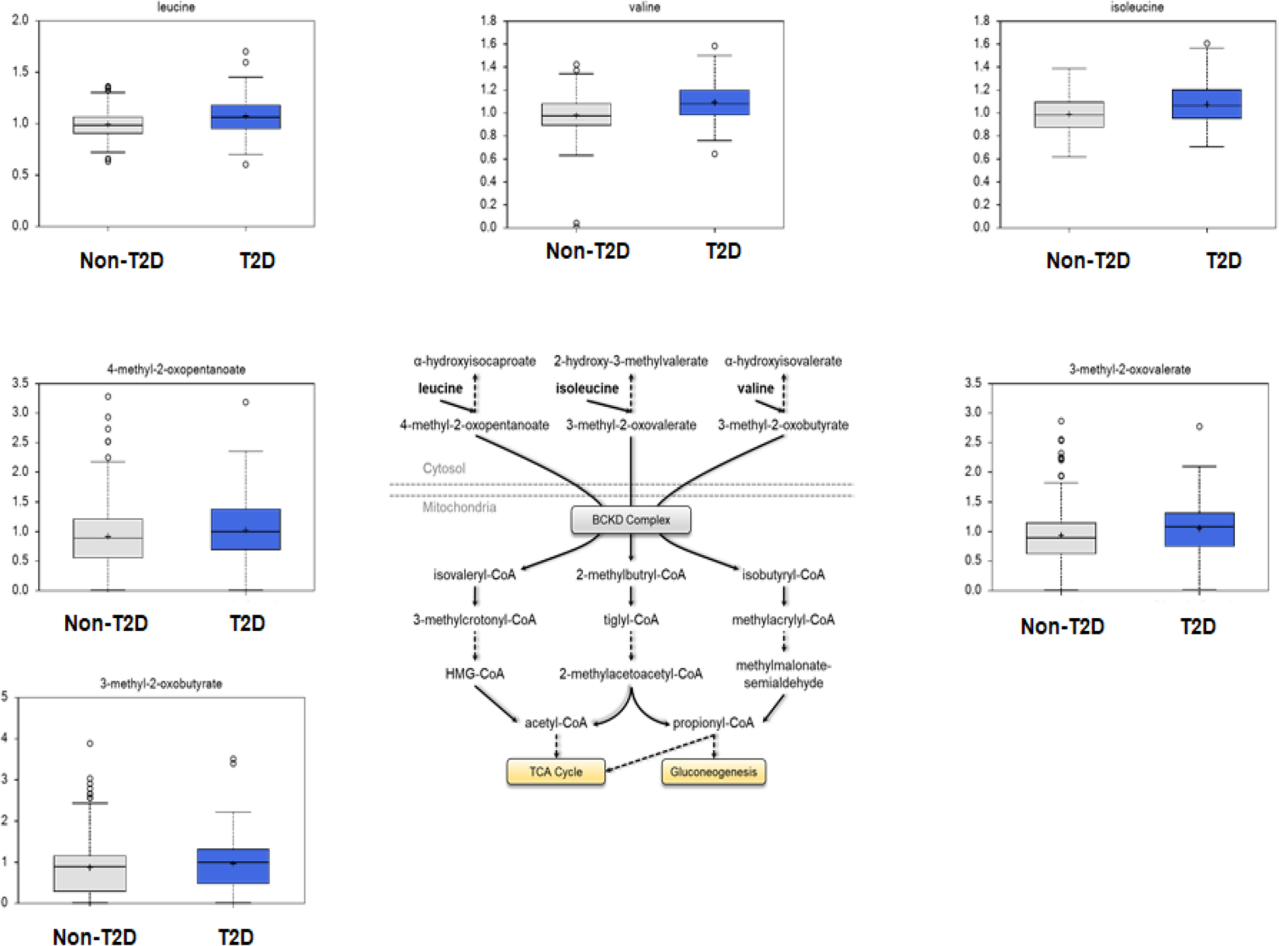
Box plots of differentially expressed branched chain amino acids (BCAA) and associated changes in key metabolites of BCAA catabolism. Top panel represents the most significantly increased BCAA in individuals with T2D vs. without T2D (left to right: leucine, valine, and isoleucine). Lower panel represents changes in intermediates and downstream metabolites in BCAA catabolism and the diagram of BCAA catabolism

Aliphatic amino acid derivatives such as N-methyl proline and N–N-dimethylalanine were decreased in T2D (Table 3) while branched-chain amino acids (BCAA) leucine, isoleucine, and valine were significantly higher in individuals with T2D than in those without T2D (Fig. 5). High plasma levels of BCAA could reflect dietary intake or muscle protein catabolism. Alongside these BCAA changes, we observed higher levels in T2D cases compared to controls of metabolites, mainly keto-acids, found downstream of the BCAA in their catabolism pathways: 4-methyl-2-oxopentanoate, 3-methyl-2-oxovalerate, and 3-methyl-2-oxobutyrate. Other catabolic BCAA products including the C2/3 and C5 acylcarnitines (e.g., propionylcarnitine, 2-methylbutyrylcarnitine and isovalerylcarnitine) were not increased in T2D (Additional file 4 (Fig S2) and Additional file 2 (Table 2D)), indicating that only a subset of products of BCAA catabolism are increased in T2D.

## Identification of a T2D metabolic signature

**Fig. 6:**
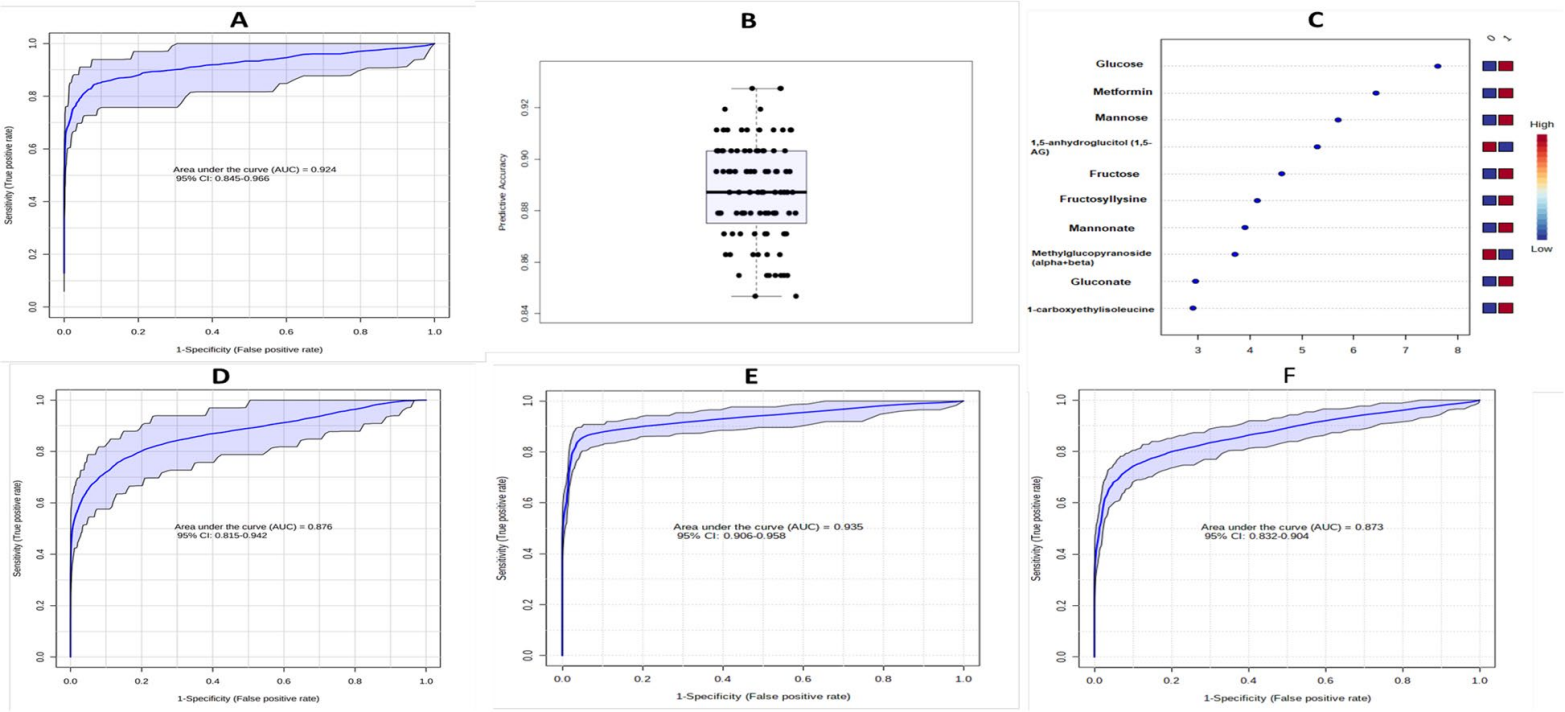
Analysis of biomarker panels for T2D based on ROC curve analyses. **A** ROC curve for the 10-metabolite biomarker panel in the discovery cohort. **B** Box plot of the predictive accuracy of the 10-metabolite biomarker panel in the discovery cohort. **C** Plot of the most important features of the 10-metabolite biomarker panel; 0 = non-T2D (individuals without T2D), 1 = T2D (individuals with T2D). **D** ROC curve for the 7-metabolite biomarker panel in the discovery cohort (panel restricted to non-established biomarkers). **E** ROC curve representing the replication of the identified biomarker panel in a different set of participants (replication cohort). **F** ROC curve representing the evaluation of the panel restricted to the non-established biomarkers in a different set of participants (replication cohort)

To identify biomarkers that can classify T2D cases and controls, we used random forest analysis followed by a multivariable exploratory ROC curve analysis with automated feature selection (Additional file 4 (Fig S3)). We found that a biomarker model consisting of 10 metabolites outperformed all other models with AUC = 0.924 (95% CI: [0.845–0.966]) (Fig. 6A) and an overall predicted average accuracy of 89.3% (Fig. 6B, Additional file 4 (Fig S4). In addition to expected classifying metabolites (such as glucose and metformin), the metabolites in the importance plot (Table 4, Fig. 6C) included several carbohydrates (mannose, 1,5- anhydroglucitol, and fructose) that were among the most differentially expressed metabolites between T2D cases and controls. Amino acids and xenobiotics were also among the biomarkers identified in this study (Table 4, Fig. 6C). Eight out of the 10 metabolites in the biomarker panel were higher in individuals with T2D compared with those without T2D (Fig. 6C). Two of the biomarkers, glucose and 1,5-anhydroglucitol, are established T2D biomarkers. In a sub-analysis, we removed from the panel of 10 metabolites metformin (because this drug/xenobiotic will not always be the treatment for all T2D cases), glucose (a diagnostic marker of T2D) and 1,5- anhydroglucitol (an established biomarker of T2D) and reassessed the discriminatory power of the restricted 7-metabolite panel (Table 4). The restricted panel had an AUC of 0.876 (95% CI: [0.815–0.942]) and a predictive average accuracy of 85.4% (Fig. 6D), showing that this panel of novel biomarkers of T2D that omits glucose (a diagnostic biomarker of T2D) can be a sufficiently useful classification tool.

**Table 4:** Metabolites in the T2D biomarker panels

	**Original (10-metabolite) panel**		**Restricted (7-metabolite) panel**	
	**Discovery**	**Replication**	**Discovery**	**Replication**
	1,5-anhydroglucitol (1,5-AG) Methyl glucopyranoside (α + β) Fructosyl-lysine Gluconate Fructose 1-carboxyethylisoleucine^a^ Mannonate Glucose Mannose Metformin	1,5-anhydroglucitol (1,5-AG) Methyl glucopyranoside (α + β) Fructosyl-lysine Gluconate Fructose Mannonate Glucose Mannose Metformin	Methyl glucopyranoside (α + β) Fructosyl-lysine Gluconate Fructose 1-carboxyethylisoleucine^a^ Mannonate Mannose	Methyl glucopyranoside (α + β) Fructosyl-lysine Gluconate Fructose Mannonate Mannose
**AUC**	0.924	0.935	0.876	0.873
**Predictive accuracy (%)**	89.3	88.8	85.4	79.5

^a^Metabolite not detected in replication cohort samples

In the replication study, we evaluated the performances of the 10-metabolite and 7-metabolite panels in an additional 270 participants from the AADM study using the same methods that we used in the discovery phase. Of the 10 metabolites present in the identified biomarker panel, 9 were available for evaluation while one (carboxylethylleucine) was not detected in the replication cohort (Table 4). Therefore, we evaluated panels of 9 and 6 metabolites in this analysis. The 9- and 6-metabolite panels effectively classified T2D cases and controls with an AUC of 0.935 (95% CI: [0.906–0.958]) and 0.873 (95% CI: [0.837–0.909]), respectively, (Table 4, Fig. 6 E, F) with average predictive accuracies of 88.8% and 79.5% (Additional file 4 (Fig S5). Similar to the findings in the discovery phase, most metabolites were increased in T2D cases compared to controls (Additional file 3 (Table S4B)).

### Correlation of the biomarker panel with clinical indices of glycemic status

**Fig. 7:**
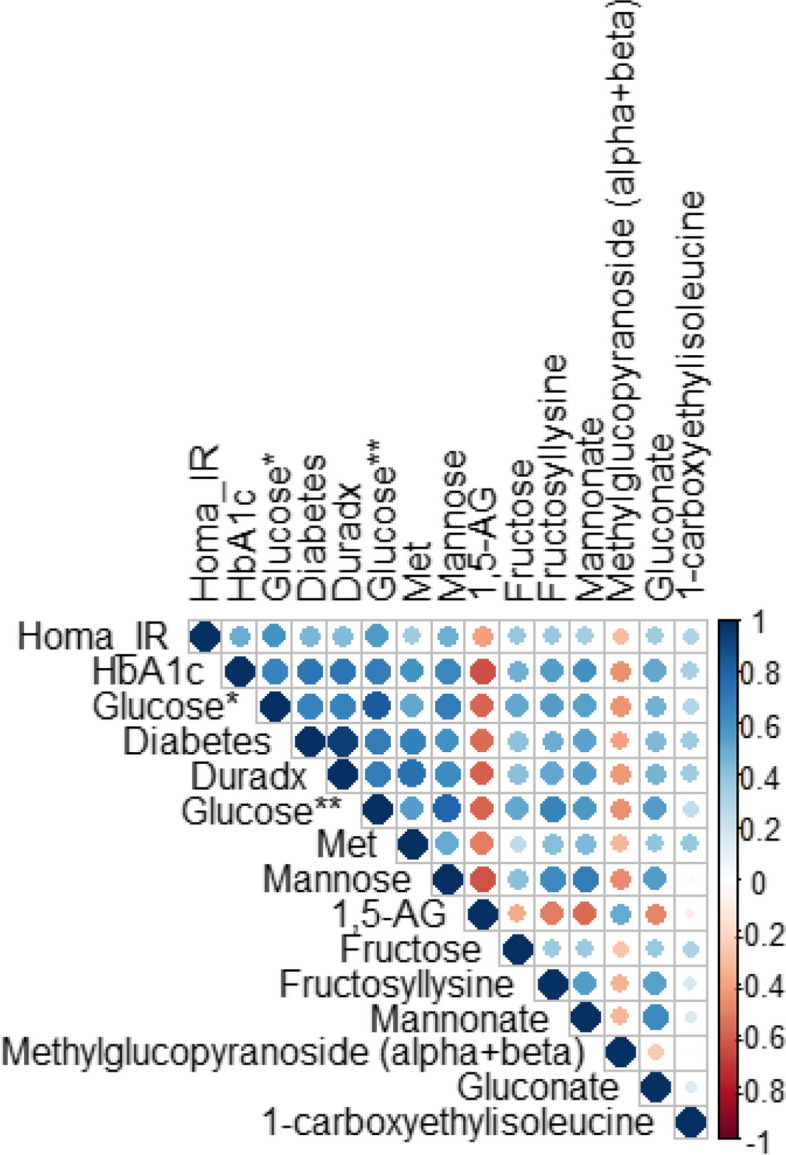
Spearman correlation matrix between metabolites in the biomarker panel and clinical indexes of type 2 diabetes in the merged cohorts (discovery + replication). *Glucose measured as part of the biochemical panel. **Glucose measured as part of the untargeted metabolomics

Given that the identified biomarker panels classified T2D cases and controls with comparable performance in both the discovery and replication cohorts, we merged the two cohorts (*N* = 580) to assess the correlation between the metabolites in the panel and several indices of glycemic status, including HbA1c, insulin resistance (HOMA-IR), and duration of T2D. As expected, glucose was positively correlated with clinical indices (0.57 < r ≤ 0.70) while 1,5 anhydroglucitol was negatively correlated (-0.64 < *r* < -0.42). Like glucose, mannose was positively associated with the glycemic indices (0.48 < r ≤ 0.69) (Fig. 7). The metabolites in the biomarker panel were moderately correlated with the markers of glycemic status but showed moderate to high correlations with each other. The strengths of the associations were more pronounced between blood sugars and their derivatives (r_(glucose/mannose)_ = 0.80, *p* < 0.0001; r_(mannose/mannonate)_ = 0.69, *p* < 0.0001; r _(glucose/ fructose)_ = 0.52, *p* < 0.0001; r _(glucose/ gluconate)_ = 0.56, *p* < 0.0001) (Fig. 7). Eight of the ten metabolites in the panel were positively correlated with T2D duration (Fig. 7).

### Effect of treatment on metabolomic profile among T2D cases

To evaluate the effect of treatment in normalizing the observed metabolic dysregulation in T2D patients, we divided individuals with T2D in this study (*N* = 260) into two groups based on HbA1C per the ADA guidelines (< 7%is controlled T2D (*N* = 102) and ≥ 7% is uncontrolled T2D (*N* = 158)) (Additional file 1, Table S5). Using ANOVA, we compared metabolites concentrations between controlled T2D cases, uncontrolled T2D cases, and individuals without T2D and used hierarchical clustering to visualize the changes between groups (heatmaps). The underlying hypothesis in this analysis is that if the metabolic profile of the controlled T2D group is similar to the profile of individuals without T2D rather than the uncontrolled T2D group, treatment has an effect in normalizing metabolic dysregulation. As shown in the heatmap figures (Additional file 4, Fig S6), across the 30 top ranking DEMs, T2D cases in the controlled group had an intermediate metabolic profile between the uncontrolled group and that of individuals without T2D. This profile suggests that treatment normalizes but does not fully correct the metabolomic dysregulation observed in T2D in our study.

## Discussion

Plasma metabolomics have been studied in many populations to understand the pathophysiology of metabolic disorders, including T2D [36, 39, 62–64]. Motivated by the need to better understand the molecular dysregulation associated with T2D in Africans, we conducted an untargeted metabolomics study using state-of-the-art high-throughput methods. To our knowledge, this is the first study to use an untargeted metabolomic approach to evaluate metabolomic profiles and analyze metabolic signatures of T2D in a large population of Africans. A key finding was the identification of 280 DEMs for T2D, implying widespread metabolic dysregulation associated with T2D. The DEMs overwhelmingly belong to the super pathways of lipids, amino acids, carbohydrates, and xenobiotics while sub-pathway analysis showed that glycolysis, free fatty acid and bile metabolism, and branched chain amino acid catabolism were dysregulated in T2D. These observations further reinforce the concept of T2D as a multisystemic disorder with a complex pathophysiology, not just a disorder of glucose metabolism. Another important component of our study was a biomarker analysis that successfully identified and validated a panel of metabolites that was able to distinguish T2D cases from controls with a high predictive accuracy of ~ 89% and AUC greater than 90%.

Consistent with other metabolomic studies, we confirmed that metabolism of free fatty acids (FFA) may be implicated in the pathogenesis of T2D [38, 63, 65]. Like others, we found that FFA (such as palmitate and stearate) were elevated in T2D individuals compared to those without T2D, but we also found that upstream products of FFA in the lipolysis pathway including mono- and di-acylglycerols were significantly increased in individuals with T2D, suggesting increased lipolysis in T2D [38, 63, 65]. Interestingly, the serum stearate/palmitate ratio is a potential predictor of diabetes remission in Chinese individuals after bariatric surgery [66]. FFA that are classified as medium chain fatty acids and saturated (i.e., consisting of 16 C or greater) have been shown to be cytotoxic to pancreatic beta cells and to affect insulin secretion [67]. High circulating FFA (especially saturated FFA), as seen in this study, are believed to inhibit insulin signaling in the muscle, possibly by reducing GLUT4 expression [68]. In contrast, polyunsaturated FFA are less toxic to and do not induce apoptosis of beta cells and were overall lower in T2D cases in our study [67].

In healthy states, the major sources of circulating FFA, adipocyte lipolysis and de novo FFA synthesis, are tightly regulated and controlled by glucose metabolism [69]. For example, FFA are increased in the fasted state but can also increase due to insufficient peripheral insulin action to suppress adipocyte lipolysis [70] as seen in insulin resistance. In our study, given that all participants were in fasted state, we can infer that the differences seen in circulating FFA between individuals with and without T2D are more likely due to the ineffectiveness of insulin to suppress lipolysis due to insulin resistance as shown by the observed high HOMA-IR and 2-hydroxybutyrate in T2D participants. 2-hydroxybutyrate, or its conjugate base α- hydroxybutyrate, is an early marker of impaired glucose regulation and insulin resistance, with a mechanism that possibly involves increased lipid oxidation and oxidative stress [71].

For cells to use fatty acids for energy, fatty acids must be transported across the cell membrane. The enzyme carnitine palmitoyl transferase (CPT1) exchanges carnitine for CoA on fatty acids to generate acylcarnitines and thus permit the movement of acyl-chains across the mitochondrial membrane to facilitate fatty acid β-oxidation [72]. When cellular free fatty acids are in excess of the cells ability to utilize them in β-oxidation or complex lipid assembly, acylcarnitines can cross the cellular membrane to be exported to the bloodstream [72]. Previous studies in African American women with T2D reported higher levels of short chain acyl carnitines, suggesting that these changes reflect incomplete fatty acid β-oxidation [73, 74]. In this study, we found no evidence of decreased or incomplete β-oxidation as shown by the lack of significant difference in short chain acyl carnitines. However, a marker of ketoacidosis, 3-hydroxybutyrate or β- hydroxybutyrate (BHBA), trended higher in T2D cases, suggesting inability of the cells to produce enough oxaloacetate (which is derived from pyruvate during glycolysis) to pair with the available acetyl-CoA generated from FFA β-oxidation to enter the tricarboxylic cycle [75]. An oxaloacetate deficiency, combined with excess acetyl-CoA, shifts the metabolism of acetyl-CoA towards ketone body formation [75]. We observed a nominally higher level of lactate in T2D cases compared to controls, suggesting increased non-oxidative glycolysis (conversion of pyruvate into lactate) associated with insulin resistance and diabetes [76]. Increased non-oxidative glycolysis could partially explain the unavailability of pyruvate to form oxaloacetate molecules needed for the TCA cycle. Other ketogenic molecules, including branched-chain amino acid BCAAs (leucine, isoleucine, and valine) and their catabolic by-products, were also higher in T2D cases compared to controls, consistent with findings from previous studies including those conducted in African Americans [74, 77–80]. Increased levels of ketone bodies, especially β-hydroxybutyrate and its intracellular derivatives, have been reported in ketosis-prone T2D (KPT2D), a form of T2D that has been often reported in African, African American, and Hispanic populations as well as in individuals on low carbohydrate diets [81]. While our findings may point to a molecular signature of KPT2D within this study, a more systematic clinical and cellular characterization of this subtype of T2D is warranted. In addition to an apparent increase of β-oxidation, ω-oxidation appears to be increased in our study. ω-oxidation is upregulated when there is increased FFA outside the mitochondria due to either increased lipolysis and/or increased dietary consumption of medium and long chain fats found in omega rich oil.

We also observed differences in bile acids composition, with both primary and secondary bile acids increased in T2D cases compared to controls. Similar observations have been made in both clinical trials and animal models [82]. Bile acids in the gut are subject to modification by the gut microbiota, which creates the secondary bile acids. Increased levels of secondary bile acids may be a reflection of higher primary bile acids, but may also reflect differences in the gut microbiota [82]. However, other amino acid-derived metabolites that are bacterial co-metabolites (e.g., cresol sulfate, phenol sulfate, phenyl lactate (PLA), and indoxyl sulfate) were not different between the groups in our study; investigating the correlation between the fecal microbiome and these markers may provide useful insights. Bile acids also play an important role in glucose metabolism through the nuclear receptor farnesoid X receptor (FXR) and transmembrane G protein-coupled receptor 5 (TGR5) [82]. Bile acid sequestrants were shown to improve glycemia in T2D patients and were approved in the United States of America for T2D treatment in 2008 [83].

Like FFA, BCAAs are associated with insulin resistance, and recent studies provide experimental evidence of interaction between BCAAs and lipid metabolism [77]. BCAA restriction in Zucker rats improves not only insulin sensitivity in skeletal muscle but also favors fatty acid oxidation [84]. Paradoxically, increased levels of BCAAs and derivative keto-acids (C3 and C5 acylcarnitines) were not increased in our study. In human studies, increased C3 and C5 acyl carnitines in plasma and muscle were associated with insulin resistance [85]. Data from the Insulin Resistance Atherosclerosis Study (IRAS) suggests that there are associations of elevated BCAAs and insulin resistance in Caucasians and Hispanics, but not in African Americans [86]. The current data lends support for ancestral differences in BCAA catabolism in individuals with T2D. Taken together, the pathophysiology of T2D at the metabolomic level appears to involve complex and tightly regulated interactions between glucose metabolism, amino acid catabolism, and lipid metabolism.

One of our goals in this study was to take advantage of the systems biology information represented by metabolomics to identify a panel of metabolites that can classify T2D individuals but also to assess the physiologic or pathologic effects of these metabolites. The metabolic signature identified in this study emphasizes impaired glucose utilization characterized by hyperglycemia and increased flux of excess glucose toward secondary conversion pathways, i.e., high mannose, fructose, mannonate, and gluconate, fructosyl-lysine, and low 1,5- anhydroglucitol. Both fructosyl-lysine (fructosamine) and 1,5-anhydroglucitol are generally a reflection of short-term glucose status, unlike hemoglobin A1c (HBA1c), which is a marker of longer-term glycemic control [87]. As previously reported, we observed an inverse relationship between glucose and 1,5- anhydroglucitol. Lower 1,5- anhydroglucitol with higher glucose is often observed in hyperglycemic subjects, due to competition between 1,5-anhydroglucitol and glucose for reabsorption in the kidney [87]. Fructosyl-lysine and its degradation by-products (advanced glycation end products (AGEs)) have been associated with vascular complications of diabetes and proposed as biomarker of diabetes complications [88, 89]. Blood sugars (1,5-anhydroglucitol, mannose, fructose, mannonate) identified in our panel were also reported in the metabolic signature of a T2D subtype known as Severe Insulin Deficient Diabetes (SIDD) in an Arab population [90]. SIDD appears to be characterized by young age of onset, low BMI, low insulin secretion, and poor glycemic control. This T2D subtype was first identified in Europeans and replicated in many other populations but not in African populations [90]. Most participants in our two cohorts are phenotypically closer to another subtype of T2D known as Severe Insulin Resistance Diabetes (SIRD) characterized by high BMI and a high level of IR [90]. The observed correlations between metabolites and clinical indices of T2D support that the pathways associated with these metabolites could be interconnected under T2D pathology. For example, high correlations between blood sugars and derivatives could be the reflection of hyperglycemia activating alternative glucose utilization pathways such as the polyol pathway, which has been associated with diabetes complications [91].

Our study has several strengths, including the use of an untargeted metabolomics approach, a relatively large sample size, inclusion of both discovery and replication cohorts, as well as the focus on an understudied population. Nonetheless, it is not without limitations. This is a cross-sectional study; therefore, we cannot infer causality. The design of the study does not allow us to categorically attribute the changes observed to T2D, its consequences, or to the use of anti-diabetic drugs. Although the sub-analysis to assess the effect of treatment on the metabolomic profile suggests that anti-diabetic drugs may partially normalize the concentrations of dysregulated metabolites, more studies are needed to understand the molecular mechanisms involved. Several identified DEMs have both endogenous and exogenous origins, i.e., diet or by-products of the gut microbiota. However, the method we used to capture metabolomics does not distinguish between endogenous and exogenous metabolites. Analyzing dietary and other omics data would help better decipher some of our findings, as would methods to infer causality (such as Mendelian randomization).

## Conclusions

In summary, this study identified profound differences in the plasma metabolic profiles of Nigerian individuals with T2D compared with those without T2D. Many of these differences, such as those in glucose, lipid, and BCAA metabolism, have been established as being involved in the pathogenesis of or secondary to insulin resistance and diabetes predominantly in populations of European ancestry. We not only successfully identified DEMs for T2D, but we also developed and validated a biomarker panel which, in addition to marking T2D status, could also be potentially useful in evaluating glycemic control, T2D duration, and T2D complications. This first study to systematically use an untargeted metabolomics approach to characterize T2D in an African population provides significant insights into the pathophysiology and heterogeneity of T2D including ketosis-prone sub-phenotype and generated global access to a critical omics dataset of Africans.

### Supplementary Information

**Additional file 1.** Description of T2D drugs and related by-products identified in the study and anthropometric/clinical characteristics of participants. The file includes Table S1A; T2D medications reported by study participants; Table S1B, T2D medications and related products identified in the study participants by LC/MS; Table S3, Anthropometric and clinical characteristics of the study participants in the replication cohort; Table S5, Anthropometric and clinical characteristics of T2D cases in the entire cohort based on controlled/uncontrolled glycemic index.

**Additional file 2.** Plasma metabolites identified in the discovery samples. It depicts comprehensive lists of all named plasma metabolites identified in the 310 participants included in the discovery phase of the study sorted by significance level, fold change, and/or by super pathway and includes Table S2A, Heat map of all 1116 metabolites identified in the discovery cohort (statistically different metabolites are sorted by FC and found on the top tier of the table whereas non statistically significant metabolites are at the bottom tiers of the table);Table S2B, Heat map of the 301 differentially expressed metabolites between individuals with T2D and withoutT2D (*p* < 0.05);Table S2C, Heat map of the 280 differentially expressed metabolites between individuals with T2D and without T2D (FDR < 0.1)**;**Table S2D**,** Heat map of the 280 differentially expressed metabolites between individuals with T2D and without T2D sorted by super pathways (FDR < 0.1). Green cells: denotes lower mean value in individuals with T2D compared those without T2D; Red cells: denotes higher mean value in individuals with T2D compared those without T2D; Light red cells and light green cells shaded cells indicate 0.05 < *p* < 0.10 (light red indicates that the mean values trend higher in T2D; light green values trend lower).

**Additional file 3.** Plasma metabolites identified in the replication samples. It depicts comprehensive lists of all named plasma metabolites identified in the 270 participants included in the replication phase of the study sorted by significance level, fold change, and/or by super pathway and includes Table S4A, Heat map of all 1071 metabolites identified in the evaluation cohort (sorted from the lowest FC to the highest FC)**;**Table S4B,Heat map of the 343 differentially expressed metabolites between individuals with T2D and without T2D (*p* < 0.05 and FDR < 0.1)**;**Table S4C**,** Heat map of the 343 differentially expressed metabolites between individuals with T2D and without T2D sorted by super pathways; Table S4D, List of shared DEMs between the discovery and the validation cohorts (141 DEMs).

**Additional file 4.** Supplementary figures supporting findings of the study. This file contains additional figures that illustrate our results and give more insights into our analyses. Fig S1, Pie chart of the different super pathways over-represented in the differentially expressed metabolites in individuals with T2D vs. individuals without T2D in the validation cohort; Fig S2, Box plots of examples of short-chain acyl carnitines in individuals with T2D and without T2D**;** Fig S3, Plot of ROC curves for all predicted biomarker model based on average performance across all MCCV runs; Fig S4**,** Predictive accuracies of all biomarker models generated using the discovery metabolomics data; Fig S5**,** Predictive accuracies of identified biomarker panels in the replication cohort; Fig S6**,** Effect of treatment on metabolomic profiles in T2D cases.

## Data Availability

The datasets generated and analyzed during the current study have been deposited in and are available from the dbGaP website, under dbGaP accession phs001844.v1.p1**.** /https://www.ncbi.nlm.nih.gov/projects/gap/cgi-bin/study.cgi?study_id=phs001844.v1.p1

## Abbreviations

AADM: Africa America Diabetes Mellitus
AGEs: Advanced glycation end products
AUC: Area under the curve
BCAA: Branched chain amino acids
BHBA: β − Hydroxybutyrate
BMI: Body mass index
Cm: Centimeter
CPT1: Carnitine palmitoyl transferase
Da: Dalton
DEMs: Differentially expressed metabolites
dL: Deciliter
FC: Fold change
FDR: False discovery rate
FFA: Free fatty acids
FXR: Farnesoid X receptor
Hba1C: Hemoglobin A1C
HILIC: Hydrophilic interaction liquid chromatography
HOMA-IR: Homeostatic model assessment for insulin resistance
IQR: Interquartile ranges
IRAS: Insulin Resistance Atherosclerosis Study
IRB: Institutional review board
Kg: Kilogram
KPT2D: Ketosis-prone type 2 diabetes
L: Liter
LC-GC: Liquid chromatography-gas chromatography
Met: Metformin
Mg: Milligram
NHREC: National Health Research Ethics Committee of Nigeria
OGTT: Oral glucose tolerance test
PLA: Phenyl lactate
QC: Quality control
RF: Random Forest
ROC: Receiver operating characteristic
(RP)/UPLC-MS/MS: Reverse phase/ ultra-performance liquid chromatography/ mass spectrometry
SIRD: Severe Insulin Resistance Diabetes
SSA: Sub-Saharan Africa
SU: Sulfonylureas
TCA: Tricarboxylic acid
TGR5: Transmembrane G protein-coupled receptor 5
T2D: Type 2 diabetes
