# Supplementary material for: Untargeted metabolomic profiling reveals molecular signatures associated with type 2 diabetes in Nigerians
Source: Genome Med. 2024 Mar 5;16:38. doi: 10.1186/s13073-024-01308-5 (PMC10913364; doi:10.1186/s13073-024-01308-5)
Supplement: Supplementary file 1 — Additional file 1. Description of T2D drugs and related by-products identified in the study and anthropometric/clinical characteristics of participants. The file includes Table S1A; T2D medications reported by study participants; Table S1B, T2D medications and related products identified in the study participants by LC/MS; Table S3, Anthropometric and clinical characteristics of the study participants in the replication cohort; Table S5, Anthropometric and clinical characteristics of T2D cases in the entire cohort based on controlled/uncontrolled glycemic index. [file 13073_2024_1308_MOESM1_ESM.docx]

**Additional file 1**

**Table S1. Reported and detected anti-diabetes therapies in individuals with type 2 diabetes in the discovery stage.**

*Table S1A. T2D medications reported by study participants.*

| Diabetes drug class | Frequency (percentage) |
| --- | --- |
| Metformin + sulfonylurea | 49 (49%) |
| Metformin only | 38 (38%) |
| Other anti-diabetes combination (including thiazolidinedione) | 5 (5%) |
| Sulfonylurea only | 5 (5%) |

**Table S1B. T2D medications and related products identified in the study participants by LC/MS**

| Drug/metabolite | % Positive in T2D |
| --- | --- |
| Metformin | 58 |
| Gliclazide (sulfonylurea) | 11 |
| Glyburide (sulfonylurea) | 11 |
| Pioglitazone (thiazolidinedione) | 9 |
| Ketopioglitazone (metabolite of Pioglitazone) | 9 |
| Hydroxypioglitazone (metabolite of Pioglitazone) | 9 |

**Table S3.** **Anthropometric and clinical characteristics of the study participants in the replication cohort**

| Variable | Overall (N=270) | T2D (n=160) | non-T2D (n=110) | p-value* |
| --- | --- | --- | --- | --- |
| AGE (years) | 59.0 (16.4) | 60.0 (15.42) | 57.02 (18.2) | 0.22 |
| BMI (kg/m^2^) | 31.54 (8.7) | 30.99 (8.3) | 32.08 (9.5) | 0.22 |
| pfm (%) | 37.65 (11.8) | 36.9 (11.5) | 38.4 (11.4) | 0.25 |
| Waist (cm) | 101.0 (14.0) | 101 (12) | 101.0 (16.0) | 0.63 |
| whratio | 0.96 (0.08) | 0.97 (0.08) | 0.96 (0.06) | 0.12 |
| GLUCOSE (mg/dl) | 97.0 (77.0) | 134.0 (116.5) | 81.0 (14.0) | <.0001^¥^ |
| Insulin (μU/mL) | 7.6 (6.4) | 8.3 (6.5) | 6.8 (6.3) | 0.03^¥^ |
| HOMA_IR | 2.1 (2.2) | 2.84 (2.8) | 1.4 (1.3) | <.0001^¥^ |
| HbA1c (%) | 6.1 (3.1) | 8.05 (4.4) | 5.4 (0.5) | <.0001^¥^ |
| CHOLL (mg/dl) | 192.5 (71.5) | 200.0 (76) | 189.0 (70) | 0.44 |
| HDL (mg/dl) | 50.6 (20.6) | 51.7 (22.2) | 49.8 (19) | 0.28 |
| LDL (mg/dl) | 118.5 (51.5) | 120.5 (54.0) | 115.5 (46.0) | 0.30 |
| TG (mg/dl) | 97.0 (51.0) | 102.0 (66.0) | 93.0 (42.0) | 0.26 |

BMI= body mass index; PFM= percentage fat mass; WHR= waist-to-hip ratio; HbA1c= hemoglobin A1c. TG= triglycerides; HDL= high density lipoproteins; LDL= low density lipoproteins, CHOLL: total cholesterol; T2D= individuals with T2D; non-T2D= individuals without T2D.

*Medians were compared using the two-sample median test

^¥^denote statistically different variables.

**Table S5. Anthropometric and clinical characteristics of T2D (controlled and uncontrolled based on HbA1C)**

| Variable | ALL (N=260) | Controlled T2D (N=102) | Uncontrolled T2D (N=158) | P-Value |
| --- | --- | --- | --- | --- |
| AGE (Years) | 60.13 (14.26) | 60.5 (14.84) | 60.02 (13.91) | 0.8 |
| BMI (kg/m2) | 31.45 (8.77) | 31.95 (9.05) | 30.73 (8.37) | 0.45 |
| PFM (%) | 38.4 (12.3) | 38.4 (11.2) | 38.35 (12.55) | 0.96 |
| Waist (cm) | 101 (14.13) | 102 (13.) | 101 (14.5) | 0.44 |
| WHR | 0.97 (0.09) | 0.96 (0.09) | 0.97 (0.08) | 0.13 |
| GLUCOSE (mg/dl) | 130.0 (91.5) | 96.0 (24.0) | 175.0 (106.0) | <.0001^¥^ |
| Insulin (uU/ml) | 8.6 (7.0) | 8.45 (7.2) | 8.8 (6.85) | 0.8 |
| HOMA_IR | 2.83 (2.84) | 1.98 (2.0) | 3.35 (3.61) | <.0001^¥^ |
| HbA1c (%) | 7.8 (3.55) | 6.1 (0.9) | 9.3 (3.8) | <.0001^¥^ |
| Total cholesterol (mg/dl) | 200.0 (74.0) | 193.5 (72) | 202.0 (72.5) | 0.37 |
| HDL-cholesterol (mg/dl) | 25.0 (25.0) | 26.0 (24.0) | 24.5 (24.5) | 0.8 |
| LDL-cholesterol (mg/dl) | 120.0 (57.0) | 119.0 (53.0) | 120.5 (60) | 0.9 |
| TG (mg/dl) | 105 (65.0) | 94.0 (37) | 112.0 (71.5) | <.0001^¥^ |

BMI= body mass index; PFM= percentage fat mass; WHR= waist-to-hip ratio; HbA1c= hemoglobin A1c. TG= triglycerides; HDL= high density lipoproteins; LDL= low density lipoproteins, CHOLL: total cholesterol

*Medians were compared using the two-sample median test

^¥^denote statistically different variables.
