# Supplementary material for: Untargeted metabolomic profiling reveals molecular signatures associated with type 2 diabetes in Nigerians
Source: Genome Med. 2024 Mar 5;16:38. doi: 10.1186/s13073-024-01308-5 (PMC10913364; doi:10.1186/s13073-024-01308-5)
Supplement: Supplementary file 4 — Additional file 4. Supplementary figures supporting findings of the study. This file contains additional figures that illustrate our results and give more insights into our analyses. Fig S1, Pie chart of the different super pathways over-represented in the differentially expressed metabolites in individuals with T2D vs. individuals without T2D in the validation cohort; Fig S2, Box plots of examples of short-chain acyl carnitines in individuals with T2D and without T2D; Fig S3, Plot of ROC curves for all predicted biomarker model based on average performance across all MCCV runs; Fig S4, Predictive accuracies of all biomarker models generated using the discovery metabolomics data; Fig S5, Predictive accuracies of identified biomarker panels in the replication cohort; Fig S6, Effect of treatment on metabolomic profiles in T2D cases. [file 13073_2024_1308_MOESM4_ESM.docx]

**Additional File 4**

**Fig S1. Pie chart of the different super pathways over-represented in the differentially expressed metabolites in individuals with T2D and without in the validation cohort.**

**
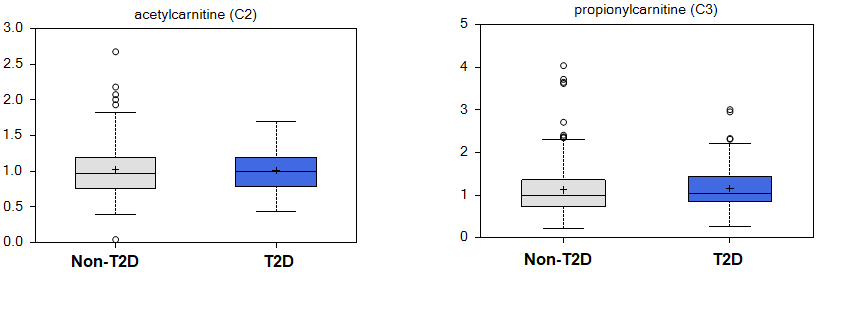
**

**Fig S2. Box plots of examples of short-chain acyl carnitines in** individuals with T2D and without T2D**.**

*****No statistically significant difference between T2D (individuals with T2D) and Non-T2D (individuals without T2D)


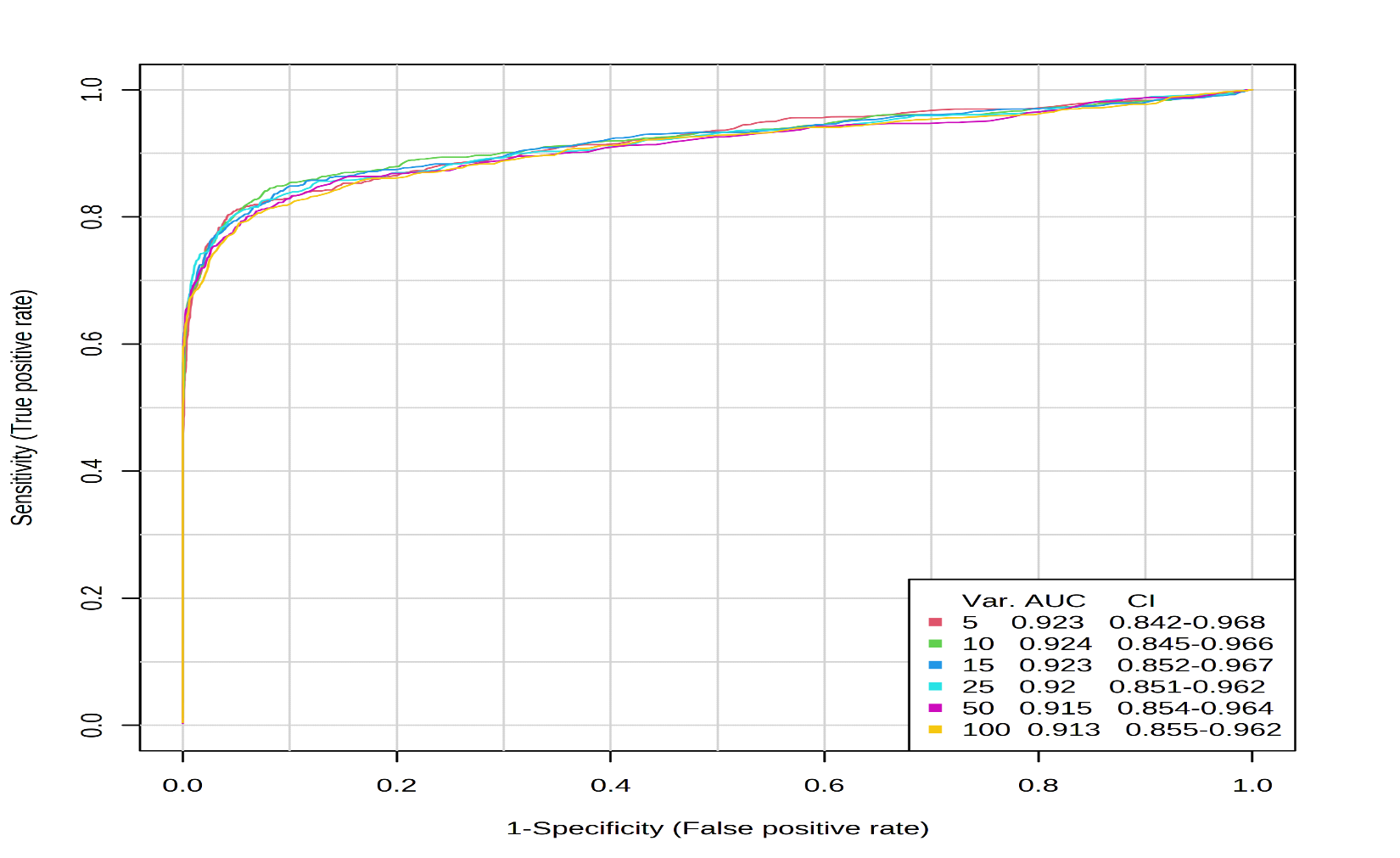


**Fig S3. Plot of ROC curves for all predicted biomarker model based on average performance across all MCCV runs**

Var.= Number of features/biomarkers in a model; CI= 95% confidence interval


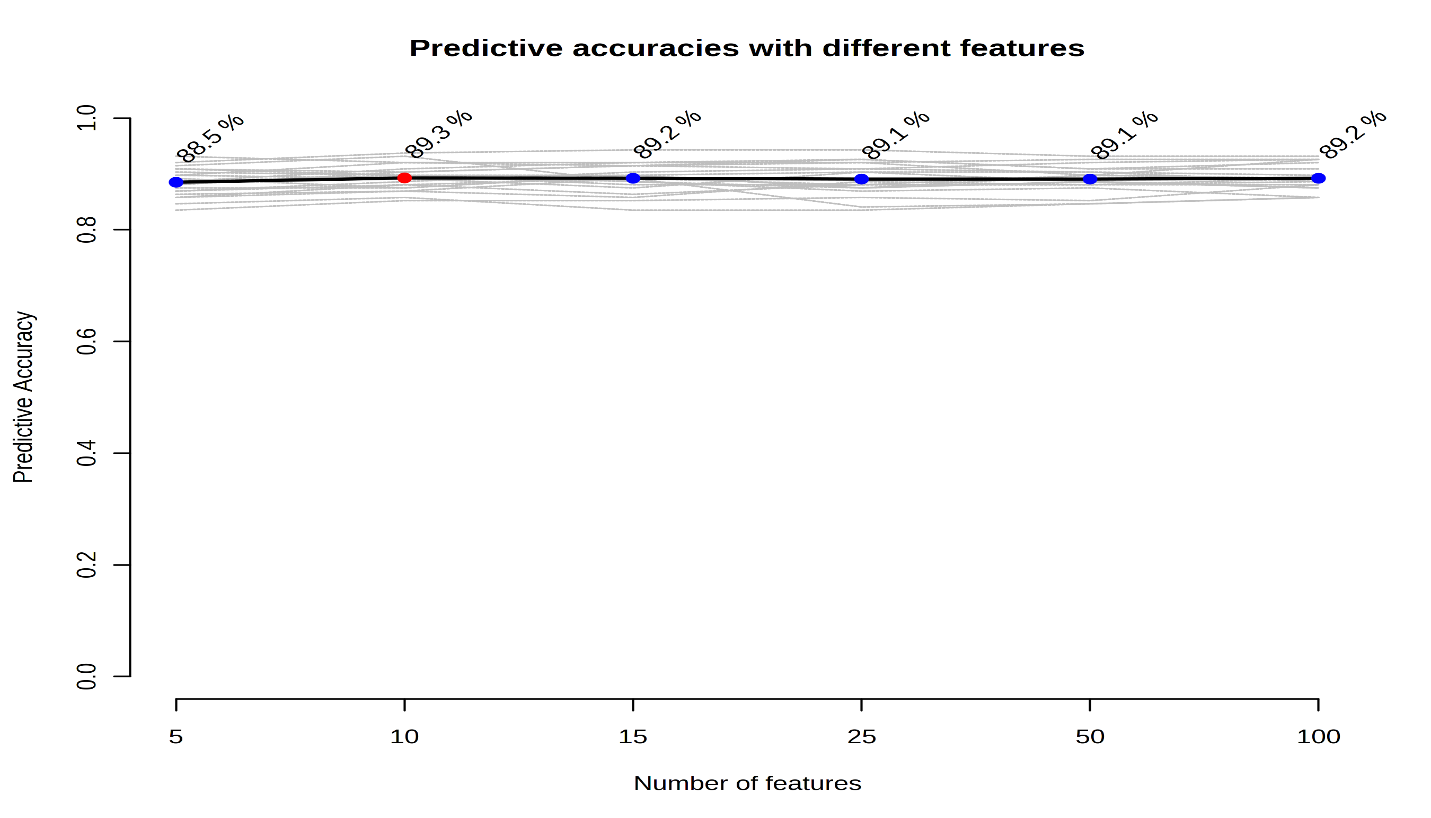


**Fig S4. Predictive accuracies of all biomarker models generated using the discovery metabolomics data.**

Red rectangle indicated the biomarker model with the highest predictive accuracy and model retained.


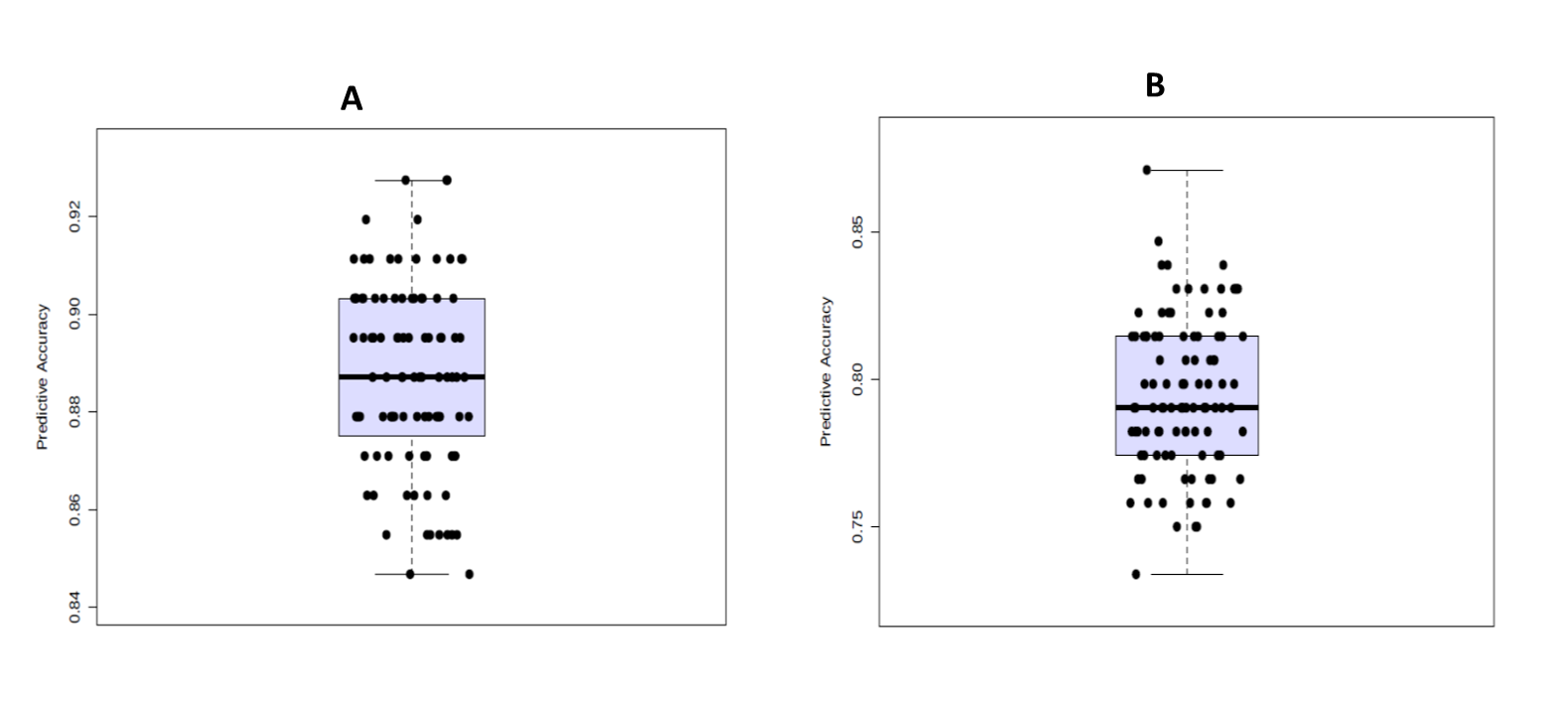


**Fig S5. Predictive accuracies of identified biomarker panels in the replication cohort**

1. Box plot of the predictive accuracy (PA) of the 9-metabolite biomarker panel: PA=88.8%. (B) Box plot of the predictive accuracy (PA) of the 6-metabolite biomarker panel (panel restricted to novel biomarkers): PA=79.5%


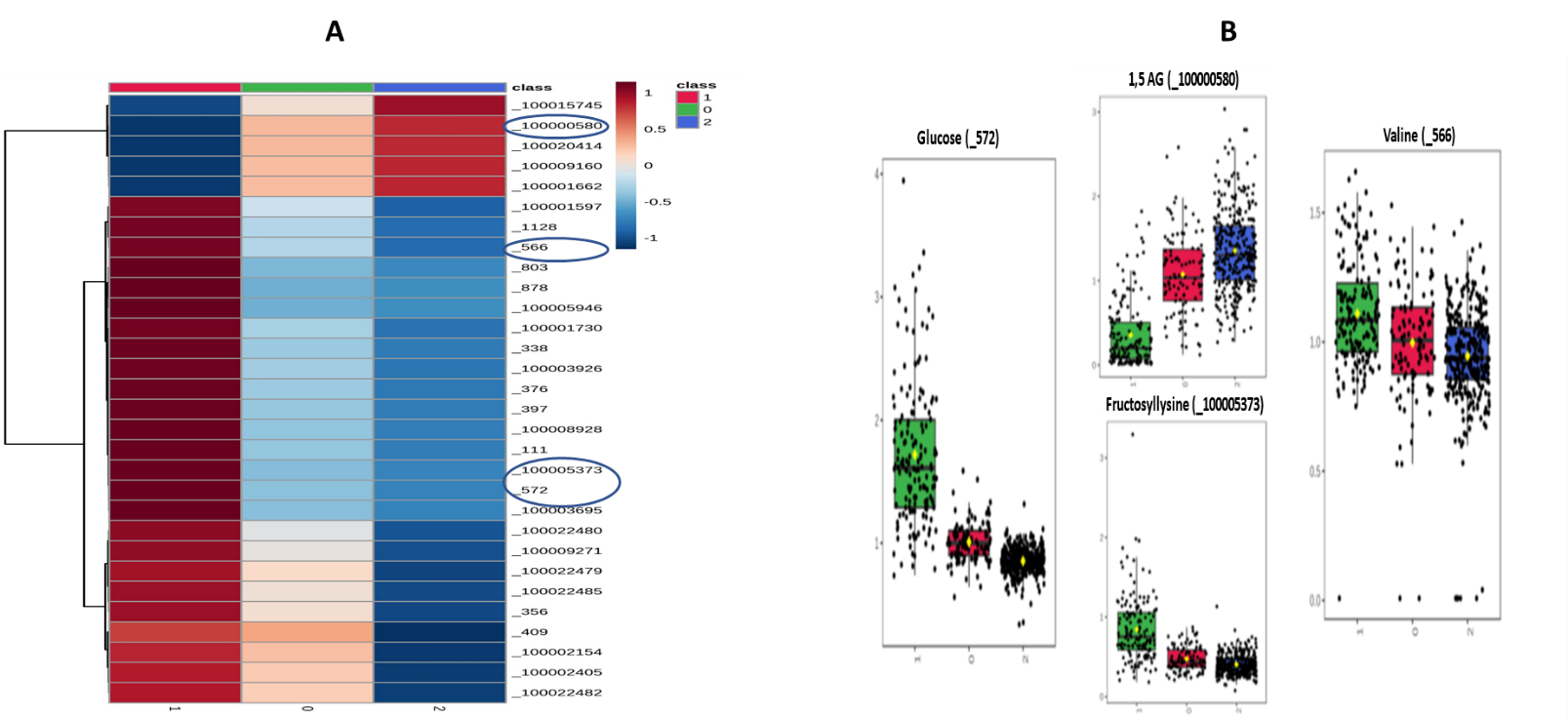


**Fig S6. Effect of treatment on metabolomic profiles in T2D cases**

1. Heatmap of the 30 top-ranking DEMs in Uncontrolled T2D (1), controlled T2D (0), and individuals without T2D) (2). concentrations of a few metabolites (circle in blue on heatmap) are displayed in (B)
2. Metabolite concentrations by group.
